# Musical Neglect Training for Chronic Persistent Unilateral Visual Neglect Post-stroke

**DOI:** 10.3389/fneur.2019.00474

**Published:** 2019-05-08

**Authors:** Kyurim Kang, Michael H. Thaut

**Affiliations:** ^1^Music and Health Science Research Collaboratory (MaHRC), Faculty of Music, University of Toronto, Toronto, ON, Canada; ^2^Collaborative Programs in Neuroscience (CPIN), University of Toronto, Toronto, ON, Canada; ^3^Rehabilitation Science Institute, University of Toronto, Toronto, ON, Canada

**Keywords:** Musical Neglect Training (MNT), Neurologic Music Therapy (NMT), unilateral visual neglect, stroke, spatial perception and attention

## Abstract

Unilateral visual neglect from right hemispheric stroke is a condition that reduces a person's ability to attend to and process stimuli in their left visual field, resulting in neglect and inattention to the left side of their environment. This perceptual processing deficit can negatively affect individuals' daily living which in turn reduces functional independence. Musical Neglect Training (MNT) has been developed based on previous research evidence to improve left visual field processing. Two individuals with persistent chronic unilateral visual neglect participated in this study. Participants underwent six individual MNT sessions. Active MNT was used involving exercises on musical equipment (tone bars) to complete musical patterns emphasizing attentional focus toward the neglect visual field. Two standardized assessments (Albert's and Line Bisection Test) were used. The assessments were administered immediately before and after each of the 6 MNT sessions to assess the within-session effect of MNT. Follow-up testing was done 1 week after their 6th session to examine the longer-lasting effects of MNT. Paired *t*-test and Wilcoxon signed rank test were used to examine results. Both participants showed significant improvement pre vs. posttest on the Albert's Test but not on the Line Bisection Test. The current study presents the positive potential of MNT for patients with chronic persistent visual neglect. In particular, effects were shown for exploratory visuomotor neglect (Albert's test), but not for egocentric perceptive neglect (Line Bisection Test), and substantiated for within-session effects only. The predictable auditory stimulus patterns associated with object sequences (tone bars) to provide feedback, direct spatial attention and orientation, and initiate intention for movement into the neglect field may offer specific advantages to reduce persistent perceptual attention deficits.

## Introduction

Unilateral visual neglect caused by right hemispheric stroke is a condition that reduces a person's ability to attend to and process stimuli in the left visual field. Research has shown that the right posterior parietal cortex (e.g., inferior parietal lobe or temporoparietal junction) (1–3), the right temporal gyrus (4), or the right frontal lobe (5) can be associated with neglect. This can negatively affect individuals' daily living tasks such as eating, grooming, writing, and getting dressed which in turn reduces functional independence. Azouvi et al. (6) reviewed and classified a number of different rehabilitation techniques and treatments for visual neglect based on theoretical basis, such as top-down mechanism (e.g., visual scanning training, visuomotor imagery therapy, and feedback of eye movements adapted glasses with auditory signals), bottom-up mechanism (e.g., prism adaptation, arm activation training, and optokinetic stimulation), modulation of inhibitory processes [e.g., transcranial magnetic stimulation [TMS] and transcranial direct current stimulation [tDCS]], and increasing arousal (e.g., sustained attention training). However, many of the existing techniques have not demonstrated significant clinical effectiveness (7, 8), and it is hard to conclude which specific rehabilitation works the best for individuals with visual neglect (6).

Music has been used as a therapeutic tool in a variety of settings to promote cognitive functions, such as memory, attention, psychosocial, or executive functions (9). For example, a study by Särkämö et al. (10) demonstrated that listening to music can help to improve positive emotion and cognitive attentional functions in participants with stroke by comparing between a music listening group and an audiobook listening group. Other studies have also shown positive effects of music on attention in older adults (11, 12), and in traumatic brain injury rehabilitation (13, 14). Furthermore, tracking and responding to repetitive auditory stimuli is part of the widely used and well-researched Attention Process Training by Sohlberg and Mateer (15). Importantly, patients with stroke anecdotally reported high levels of engagement with rehabilitation exercises that are embedded within a musical context (16).

Some earlier studies have suggested that auditory stimuli and music may have a beneficial effect on improving attention for patients with visual neglect. Robertson et al. (17) has shown that auditory stimuli can activate the right hemisphere in the brain which is dominant for sustained attention and that those stimuli can influence spatial attention including unilateral visual neglect. Research has also shown that musical stimulation may be able to improve attention and perception in the left visual field (18).

Based on those early data, Musical Neglect Training (MNT) has been developed for patients with unilateral visual neglect (19, 20). MNT uses active musical exercises rather than receptive music listening. Exercises are structured in pitch, time and tempo, and using musical equipment (tone bars, keyboards, drums, etc.) whose physical setup is configured to focus active attention to the neglect field. In the exercises, participants are asked to complete musical (melodic or rhythmic) patterns on musical instruments that are increasingly extended into the visual neglect field. Patterns should be well-known to the participants (such as familiar melodies or octave scales) to drive the attentional search to find and complete all musical events via active playing (21). This structure is intended to take advantage of the inherent perceptual structure of sequence completion in musical patterns and thus facilitate the spatial orientation and exploration toward the neglect side. It is commonly agreed upon that visual neglect is characterized by deficiencies in two separate neuropsychological processes: the perceptual-cognitive “where” construct and a premotor-intentional “aiming” component (2, 8). The combination of spatial orientation and motor execution in the perceptual and physical setup of MNT may be considered a critical mechanism in MNT since it simultaneously addresses both critical processes effectively together.

However, only two recent clinical studies have applied active MNT protocols while demonstrating positive outcomes to reduce neglect. Bodak et al. (22) examined the effectiveness of MNT using musical instruments, chime bars, which were aligned horizontally extending into the neglect visual field. Bernardi et al. (23) used playing a music scale on the keyboard with decreasing range into the neglect field. Both studies tried to exercise perceptual-attentional and motor-intentional deficits by asking subjects to complete musical patterns whose lengths was increasingly extended into the visual neglect field. While most studies have looked at subacute or short-term chronic stages post stroke (up to 20 months), only Bodak et al. (22)'s study looked at two patients with chronic persistent neglect, 5.11 and 4.5 years post stroke. Therefore, this study was intended to add to the currently limited data base of MNT in chronic persistent neglect and also to investigate carry-over effects from training 1 week after training completion.

## Description of Instrumentations and Musical Neglect Training Procedure

The Albert's Test and the Line Bisection Test, which are highly used to assess neglect (24), served as test measures in this study. In the Albert's Test subjects are required to cross out 40 black lines that are randomly orientated on a sheet of paper. The test paper was placed on a table and presented in front of a patient, parallel to their midsection. The experimenter asked the participant to cross out all of lines they see in front of them. Patients had a maximum of 5 min to complete the activity Assessment tools to be used for this test included an A4 sheet, with 40 lines 2 centimeters in length and a pencil (25). In terms of the reliability and the validity for Albert's test, excellent test-retest reliability has been reported with a score of *r* = 0.79 (26). Moreover, Albert's Test has excellent correlations with the Line Bisection Test (*r* = 0.85) (27).

In the Line Bisection subjects must place a mark with a pencil in the center of horizontal lines. A displacement of the mark toward the side of the brain lesion (the deviation from the true central point of each horizontal line) is assessed as a symptom of neglect. The equipment for this test is an A4 sheet with 17 vertically staggered horizontal lines and a pencil (28). Bailey et al. (29) reported the intraclass correlation coefficient was excellent for neglect patients (ICC = 0.97).

The Albert's Test was scored as the number of uncrossed lines on the sheet. The test paper consisted of 18 lines on each side, and 4 lines in the center. The Line Bisection Test was calculated as percentage of the deviation from the true center of the line. There were 17 horizon lines, which had different length and calculated by the following methods: the means of the deviations of all 17 lines were divided by the center point length of all 17 lines/17 and shown as percentage. The formula was [Sum of all deviations/Sum of all center point length of 17 lines)/17].

A pre-and post- test design was used to assess responses to the intervention as within-session effects. A follow-up test after 1 week was administered to determine longer-lasting effects.

The means and standard deviations were calculated for both Albert's and Line Bisection test. Paired sample *t*-tests were calculated to examine significant differences and compare means by subtracting pre- intervention scores from post- test scores. Nonparametric statistics (Wilcoxon sign-ranked test) was also calculated in parallel with the paired *t*-tests due to the small sample size and possible violations of normal distribution. For the follow-up test, the follow-up score was compared with the mean and 95% confidence interval of pre-test scores to examine a beneficial longer-lasting effect.

**Figure 1:**
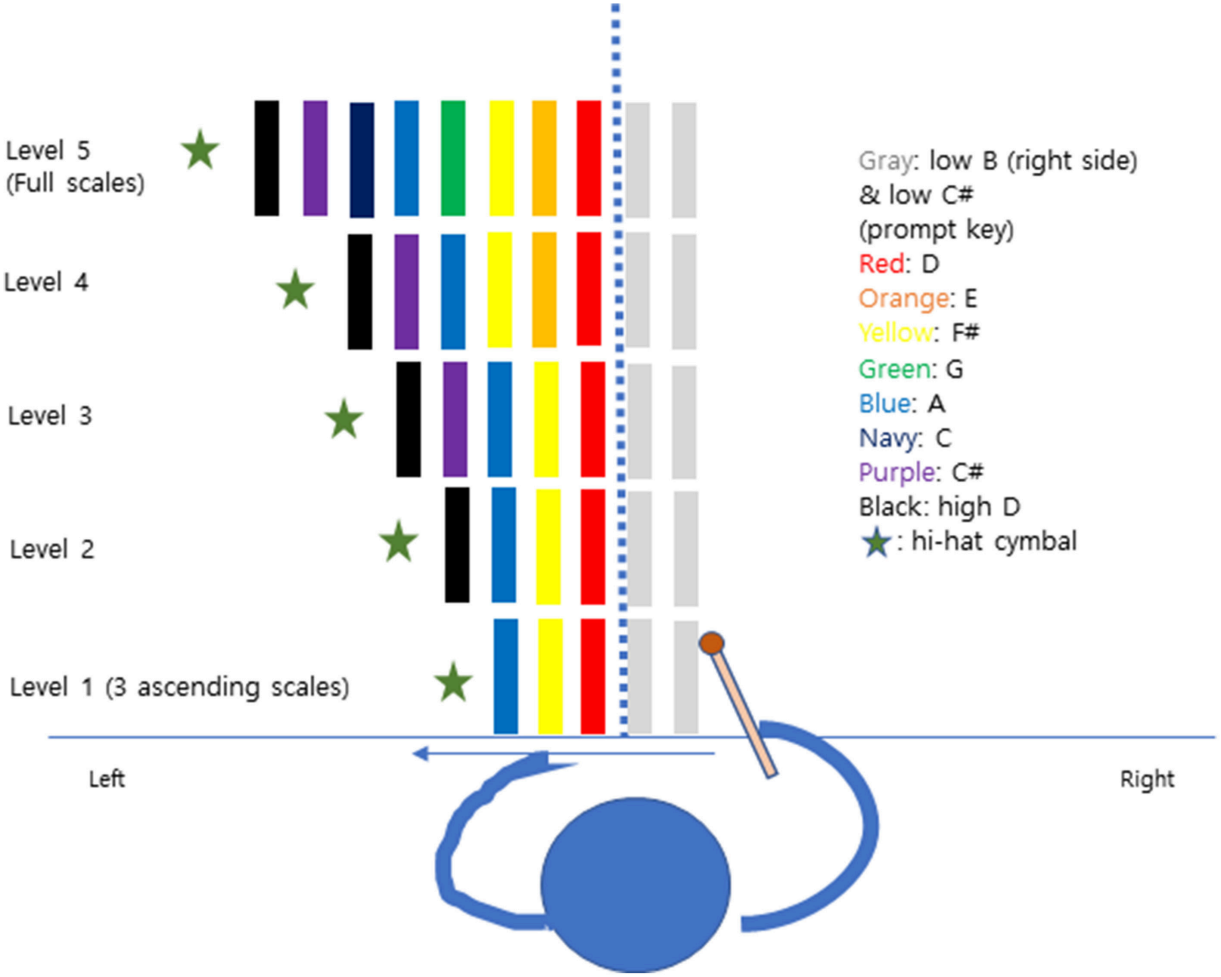
The five levels of musical neglect training.

There were 2 weekly 30 min individual sessions over a period of 3 weeks for a total of six MNT sessions per participant. The participants started with the Albert's Test and the Line Bisection Test for assessment before training. Participants sat comfortably in a chair, playing the tone bars on the desk with the non-paretic arm (right). In each level, the first tone bar (D) was put in the center of participants aligned with the midsection of the face. Also, two tone bars (B, C#) were set to the right side before starting the scale or triad to initiate the playing movement from right to left in the healthy visual field. Participants followed five protocol levels by playing 3 ascending scale to full scale (Figure 1). A hi -hat cymbal was located in the last position to give a strong sound target for completion of the pattern. In each pattern, the cymbals' edge matched the end of the very last tone bar's edge. The experimenter was positioned on the patient's non-neglect side (right) to give instruction and play a chordal keyboard accompaniment for each pitch. Participants repeated each pattern 5 times before moving to the next step. The Albert's test and the Line Bisection test were given to participants after each training session to observe within-session effects.

## Case Presentation

All participants gave written informed consent for both participation in the study, and publication of the data in accordance with the Declaration of Helsinki. This study was approved by the Institutional Review Boards of Colorado State University (14-5432H) and registered at ClinicalTrials.Gov (NCT03516825). Two participants with right hemisphere stroke who were diagnosed with a neglect syndrome were recruited for this study. Both participants met the following criteria: (1) right handed, (2) medically stable, (3) no previous music therapy experiences, (4) no hearing impairments, and (5) no cognitive deficits. Exclusion criteria were (1) hemianopia, and (2) previous music therapy treatment experiences.

### Participant 1

Participant 1 was a 62 years old female who sustained an ischemic stroke 26 month prior to entering the study. Her stroke was located in the right internal capsule and medial right temporal lobe resulting in left hemiparesis and loss of attention on the left side with decreased functional performance in activities of daily living.

Participant 1 reported that she felt left facial and left upper extremity weakness before she was admitted to the hospital. She had a history of hypertension, type 2 diabetes, depression, restless leg syndrome, and breast cancer. Her family history indicated that both her parents had a stroke. She described her visual field saying that she seemed to lose a half side of screening the world.

#### Albert's Test

**Figure 2:**
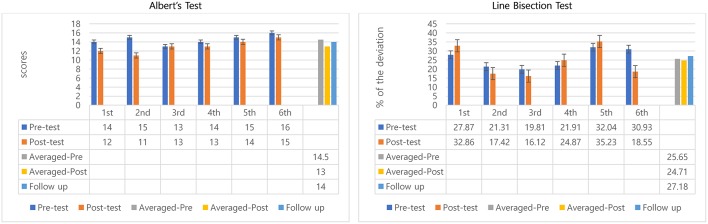
Descriptive data for participant 1's Albert's test and line bisection.

The scores were calculated as the number of uncrossed lines, so a reduced score meant improvement. All scores from pre to post-test of six sessions decreased, except at 3rd session (remained same). The mean of the scores the participant did not mark for pretest was 14.5 and 13 at post-test. Pre-test and post-test difference of the means of all 6 sessions' indicated positive outcomes regarding within-session effects—but with small differences only (14.5 to 13). Also, it is important to note that the 6th session was worse than the pre-test of the 1st session (Figure 2). A Wilcoxon signed rank test was conducted to determine whether there was a difference in the ranking of pre-and post-test by MNT sessions. Results of that analysis indicated that there was a significant difference in pre-and post-interventions, *Z* = −2.06, *p* = 0.04. Similar to the non-parametric analysis confirmed there was a significant difference in mean scores before and after MNT training (*t* = 2.67, *p* = 0.04) (Table 1).

**Table 1:** Descriptive statistics and *t*-test results for Albert's test in participant 1 and participant 2.

	**Pre-test**		**Post-test**		**Follow-up**	**Mean difference**	**95% CI**	**95% CI of Pre-test**	***t***	***df***	**Sig. (two-tailed**
	***M***	***SD***	***M***	***SD***							
P1	14.5	1.05	13.0	1.41	14	1.50	0.05, 2.95	13.66, 15.34	2.67	5	0.04^*^
P2	9.5	1.52	7.67	0.52	7	1.83	0.29, 3.38	8.29, 10.71	3.05	5	0.03^*^

* *p < 0.05*.

The average of all 6 sessions and 95% confidence intervals of pre-test scores were compared with the follow-up test score to observe the longer-lasting effect. Scores slightly decreased when compared between average of all 6 sessions' pre- (mean scores = 14.5) and follow-up test (scores = 14) (Figure 2). The follow-up test (score = 14) was inside the 95% confidence interval of pre-test [13.66, 15.34] which indicates both scores come from same populations, and, therefore, they are not significantly different (Table 1).

#### Line Bisection Test

Decreased percentage means positive effects because shorter deviations from the center point indicate better awareness of the neglect side. The percentages of deviation in all 6 sessions were inconsistent. Three sessions (2nd, 3rd, and 6th) showed improvements, but the other three sessions (1st, 4th, and 5th) did not indicate improvements. However, overall, once averaged pre-test and averaged post-test were compared, the deviation from the center declined from 25.65 to 24.17%, about a 1.5% decrease. The interesting result was that the participant showed obvious decreased percentage of deviation from 30.93 to 18.55%, about 12% decreased at 6th session. However, there was no significant difference in pre-and post-interventions (Figure 2).

For the longer-lasting effect, the follow-up test score was compared with the averaged pre-test percentage deviation of 6 sessions. Percentage of deviation in Line Bisection Test for Participant 1 increased from 25.65 to 27.18%, showing some deterioration (Figure 2). The percentage of deviation in the follow-up test (27.18%) was inside of 95% confidence intervals of pre-test [21.40, 29.89] indicating no significant difference between follow-up score and the averaged pre-test percentage deviations of the 6 training sessions.

### Participant 2

Participant 2 was a 69 years old male who had sustained stroke 10 years before entering the study. His ischemic stroke in the right parietal lobe region due to atrial fibrillation resulted in left hemiparesis, and sensory impairment in the left side. He described that he could button the buttons on the right side of his shirts without seeing but could not do this on the left side. Also, he reported that he seemed to lose a quarter to a half field of vision on the left side. However, after the initial stroke recovery and rehabilitation participant 2 had continued in a successful professional career and also reported staying active in weekly exercise engagements (biking).

#### Albert's Test

**Figure 3:**
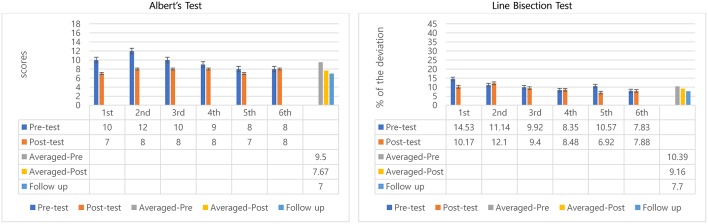
Descriptive data for participant 2's Albert's test and line bisection.

Between session 2 and 5 participant 2 showed a steady reduction in pre-test scores indicating a possible benefit transfer from session to session. Also, in sessions 1–6 all posttest scores improved over pretest except the last session (remained same), which indicated positive outcomes in within-session effects of MNT (Figure 3). Paired *t*-tests showed significant differences between pre-test (*M* = 9.5, *SD* = 1.52), and post-test (*M* = 7.67, *SD* = 0.52); *t* = −3.05, *p* = 0.03 (Table 1). Wilcoxon signed rank test confirmed averaged post-test scores to be significantly better than averaged pre-tests, *Z* = −2.03, *p* = 0.04.

Averaged pre-test scores and the follow-up test were compared to determine potential longer-lasting effect in Albert's test for participant 2 by comparing the 95% confident interval of the pre-test score with the follow-up score. Scores decreased from averaged pre-tests (mean scores = 9.5) to follow-up test (score = 7) showing improvements over pretest performance and maintenance of benefits after posttest (Figure 3). The follow-up test score was outside the 95% confident intervals of the pre-test score [8.29, 10.71] indicating the scores come from different populations, hence, they are significantly different (Table 1). This result may suggest remaining beneficial effect of MNT training at the follow-up test.

#### Line Bisection Test

Although pretest scores improved overall across sessions, the posttest outcomes measuring within-session treatment effect were inconsistent, showing only improvements in sessions 1, 3, and 5. The other sessions showed slight declines (2nd: 1%, 4th: 0.1%, and 6th: 0.05%). However, the averaged percentage of deviation from pre-test to post-test decreased about 1.2% (Figure 3). There were no significant differences between pre-and post-test in the line bisection test.

To see the longer-lasting effect, similar to the Albert's test, averaged pre-tests of 6 sessions and follow-up test were compared. Percentage deviations decreased from 10.39 to 7.70% (Figure 3). The percentage of the deviation for the follow-up test (7.70%) was outside the 95% confident interval of the pre-test's deviation percentage [8.47, 12.31] which suggests significant differences between pretests and follow-up indicating a preserved positive longer-lasting effects of the MNT intervention.

## Discussion

Taken together, participant 1 (26 months post stroke) benefitted from MNT training as evidenced by the Albert's test with statistical significance and showed small but non-significant improvements on the Line Bisection Test. Characteristic for the performance of participant 1, however, was her difficulty to sustain training benefits from session to session and at follow-up. Participant 2 (10 years post stroke) showed significant benefits from MNT training on the Albert's test with each session as well which seemed to also carry-over to reduced pretest scores from session to session, indicating that the participant's neglect status was consistently reduced across the 3-week training period. The evidence for sustaining benefits was also reflected in the follow-up score 1 week after the last training. Improvements on the Line Bisection Test were larger than for participant 1 yet too small and inconsistent to reach statistical significance. However—unlike for participant 1—the ability to carry over training benefits was also evidenced on the Line Bisection Test for this participant when comparing reductions of initial pretest scores across the next 5 sessions and the follow-up test result.

Interestingly, both participants showed significant improvements in Albert's Test, but did not show significance on the Line Bisection Test similar to results by Bodak et al. (22). In particular, participant 1 reported difficulties with the Line Bisection Test. Albert's Test focuses on finding short lines scattered around space and crossing them at no particular place. The Line Bisection Test has higher demands on horizontal and vertical perception along longer lines that have to be crossed at each midpoint. However, since each line shifts in horizontal position from each other after finding via vertical scanning the subject has to scan and re-calibrate the whole length of the line to find the new midpoint which is different than the midpoint just found on the line above. This added spatial complexity may account for the different results between the Albert's Test and the Line Bisection Test. Furthermore, this difference may be related to distinct brain regions which are associated test performances. Decreased performance on Albert's Test has been associated with dorsal and anterior lesions, while a larger deviation from the center on Line Bisection Test has been shown with lesions in parieto-occipital regions (30, 31). The evidence for the involvement of distinct brain regions may correspond with the results of our study. Participant 2, with a right parietal stroke, had demonstrated significant positive effect after MNT interventions in the Albert's test, but not the Line Bisection Test. All test scores for the Albert test of participant 2—mean pre-test and posttest and follow-up test—were better than participant 1's scores, indicating that potentially participant 2 had a less impaired attention in the left visual field than participant 1 already at onset of the study. Similar trends were shown in the Line Bisection Test, however, non-significantly.

One possible explanation is that during 10 years following his stroke, participant 2—although he never had specific therapy for his neglect—may have been working on compensatory strategies to be alert regarding his spatial unawareness of the left side in order to accommodate his daily life. On the other hand, participant 1 had a stroke 2 years ago. She also had not had any rehabilitation training for visual neglect. She mentioned that she was learning to scan her left visual field by herself for 2 years. Ten years compensatory efforts vs. 2 years may account for the different outcomes in both assessments and also account for the higher ability of participant 2 to carry over training benefits from session to session.

It has been shown in a study that followed patient's neglect functions over 12 months that they were able to anticipate their skewed spatial orientation and to compensate for neglect in structured and predictable circumstances (32). Furthermore, the severity of the neglect and brain lesions may also contribute to the different outcomes from both participants.

The current study presents the positive potential of MNT for patients with chronic persistent visual neglect in both, within-session and longer-lasting effects. Most studies have researched neglect rehabilitation with cohorts in subacute stages. The mechanisms and recovery period for visual neglect are still controversial. Chronic neglect is reported as rare, recovering mostly within 3 months post-stroke (33–38). However, research has shown that the perceptual-attention component of neglect frequently persists at the chronic stage (39–41). Furthermore, Rengachary et al. (41) found that perceptual-attention deficits showed the most variability during recovery after severe neglect states. Compared to other rehabilitation techniques and treatments for people with visual neglect (e.g., visual scanning prism adaptation, TMS, limb activation, etc.), the combination and integration in MNT of predictable auditory stimulus patterns associated with object sequences (tone bars) to provide cognitive-perceptual feedback, direct spatial attention, and orientation into the neglect side, and initiate intention for movement into the neglect field may offer specific advantages to reduce persistent perceptual attention deficits.

Furthermore, music may provide a cerebral hemispheric arousal benefit. It has been well-documented in research that musical responses and tasks activate both brain hemispheres bilaterally with a strong activation component of right hemispheric regions (42). Right parietal and visual regions have been shown to be activated during music listening in hemispatial neglect (43). Since hemispatial neglect involves a lesioned under-aroused right hemisphere the right-hemispheric stimulation in music may provide an additional neural mechanism to reduce neglect.

The two cases of chronic persistent hemispatial neglect presented here provide statistically significant evidence for MNT driving within session effects for the Albert Line Crossing test. However, several limitations need to be pointed out. To generalize the effectiveness of MNT, larger sample sizes and adjustments to the length and level of intervention would be required. Adding a multiple baseline design would also help provide more stable pre-intervention data to elucidate subsequent effects for the MNT interventions, even in single system study design. Additionally, multiple follow-up assessments (e.g., after 1 week, 3 months, and 6 months) would also provide more insight into preserving potential long-term benefits of MNT. More extended functional assessments in addition to the clinical assessments used here would also add insight into training benefits transferring to activities of daily living. Finally, investigating MNT mechanisms via employing neuroimaging techniques may shed light on the potential impact of MNT on neuroplasticity in the recovery of attention function in hemispatial neglect.

## Ethics Statement

All participants gave written informed consent for both participation in the study, and publication of the data in accordance with the Declaration of Helsinki. This study was approved by the Institutional Review Boards of Colorado State University (14-5432H) and registered at ClinicalTrials.Gov (NCT03516825).

## Author Contributions

MT contributed the design of the study procedure and statistical analysis and revised it critically for important intellectual content and final approval of the version to be submitted. KK collected the data and performed the statistical analysis and interpretation of data and wrote the first draft of the manuscript. All authors approved the submitted version.

### Conflict of Interest Statement

The authors declare that the research was conducted in the absence of any commercial or financial relationships that could be construed as a potential conflict of interest.
